# Integrating Ultra-Weak Photon Emission Analysis in Mitochondrial Research

**DOI:** 10.3389/fphys.2020.00717

**Published:** 2020-07-08

**Authors:** Roeland Van Wijk, Eduard P.A. Van Wijk, Jingxiang Pang, Meina Yang, Yu Yan, Jinxiang Han

**Affiliations:** ^1^Meluna Research, Department of Biophotonics, Geldermalsen, Netherlands; ^2^Key Laboratory for Biotech-Drugs of National Health Commission, Shandong Medicinal Biotechnology Center, Jinan, China; ^3^Shandong First Medical University, Jinan, China; ^4^Shandong Academy of Medical Sciences, Jinan, China

**Keywords:** mitochondria, reactive oxygen species, ultra-weak photon emission, stress, aging, diagnosis

## Abstract

Once regarded solely as the energy source of the cell, nowadays mitochondria are recognized to perform multiple essential functions in addition to energy production. Since the discovery of pathogenic mitochondrial DNA defects in the 1980s, research advances have revealed an increasing number of common human diseases, which share an underlying pathogenesis involving mitochondrial dysfunction. A major factor in this dysfunction is reactive oxygen species (ROS), which influence the mitochondrial-nuclear crosstalk and the link with the epigenome, an influence that provides explanations for pathogenic mechanisms. Regarding these mechanisms, we should take into account that mitochondria produce the majority of ultra-weak photon emission (UPE), an aspect that is often ignored – this type of emission may serve as assay for ROS, thus providing new opportunities for a non-invasive diagnosis of mitochondrial dysfunction. In this article, we overviewed three relevant areas of mitochondria-related research over the period 1960–2020: (a) respiration and energy production, (b) respiration-related production of free radicals and other ROS species, and (c) ultra-weak photon emission in relation to ROS and stress. First, we have outlined how these research areas initially developed independently of each other – following that, our review aims to show their stepwise integration during later stages of development. It is suggested that a further stimulation of research on UPE may have the potential to enhance the progress of modern mitochondrial research and its integration in medicine.

## Introduction

The mitochondrion is a multifunctional life sustaining organelle that is generally known as the “powerhouse of the cell” (Alberts, 2015). Except for the red blood cells that transport oxygen, each cell in the body contains hundreds to thousands of mitochondria. Recent years have seen an increase in research interest in mitochondria (Picard et al., 2016). In light of the fact that mitochondria are the only organelles that house their own genome, this rising importance of mitochondria research stems from the growing recognition that a number of medical conditions is caused or promoted by mitochondrial DNA (mtDNA) defects. Besides energy production, mitochondria produce signals that convey information between mitochondria and the nucleus including reactive oxygen species (ROS) and reactive metabolites derived from mitochondrial metabolism. Furthermore, spontaneous photon emission of unknown, if any, significance has been described from mitochondria. Ultra-weak photon emission (UPE) in the range between 300 and 900 nm, i.e., from UV to near-IR has been reported from cells, small organisms, and even human skin with fluctuating intensities (Van Wijk, 2014). A possible significance has been suggested based on increasing knowledge of low-intensity light therapy (LILT) or photobiomodulation (PBM) therapy, that uses low intensities of light with wavelengths in the visible (VIS), red, and near-IR (NIR) (Tafur et al., 2010; Kim et al., 2017). These modalities have been shown to influence a wide variety of cellular functions, including gene expression, growth and proliferation, survival, and differentiation (Shefer et al., 2002; Zhang et al., 2003). These functions are primarily mediated by raising the levels of adenosine triphosphate (ATP), a process in which cytochrome c oxidase appears to be the primary photo acceptor and transducer of photo signals in these regions of the light spectrum (Karu, 2010; Poyton and Ball, 2011). In the wavelength range important for phototherapy (330–860 nm), there are five “active” spectral regions; the generalized action spectrum for HeLa cell proliferation and DNA synthesis consists of bands with maxima at 400, 620, 680, 760, and 825 nm. The bands in the action spectrum were identified (Karu and Afanasyeva, 1995; and reviewed in Karu, 1999), allowing the conclusion that they may be related to the upregulation of cytochrome c oxidase (Karu et al., 1982, 1984; Karu and Afanasyeva, 1995; Karu, 1999). Other results with LED treatment have suggested effects on mitochondrial morphology (Kim et al., 2017). In considering this information, it is of interest to include the (yet often ignored) fact that mitochondria produce the majority of cellular UPE into the compartmentalized field of the cell biology of mitochondria consisting historically of studies on protein import, molecular genetics, bioenergetics, mitochondrial dynamics, cell death, and so on. We come to a moment in history where these islands of understanding must unite, combining also the ultra-weak photon emission in this unification because this may further advance the role of mitochondrial biology in medicine. We have also come to the moment that, next to the fundamental aspects of mitochondrial function, we may also work harder to consider mitochondrial biology in the physiology of real tissues.

Hence this review focuses on integrating UPE in mitochondrial research. Such integration is yet not completed, and therefore, this overview is presenting the current state from a historic perspective. Starting in section Three Research Areas and a Building Layer (1960–1980), our overview will first discuss the period from around 1960 onward, a period in which mitochondria-related research was initiated in three areas that, at that time, were regarded to be mutually independent: (a) respiration and energy production, (b) production of free radicals and other, non-radical ROS, and (c) UPE. Although new insights in these areas gradually lead to the realization of their interrelatedness, the actual integration of research lines has spanned several decades. In this stepwise integration process, two distinct time periods can be distinguished: (a) 1980–2000 [outlined in section A Second Layer: The First Period of Integration (1980–2000)] and (b) 2000–2020 [section Enhanced Integration: Mitochondrial Dynamics (2000–2020)].

**Figure 1:**
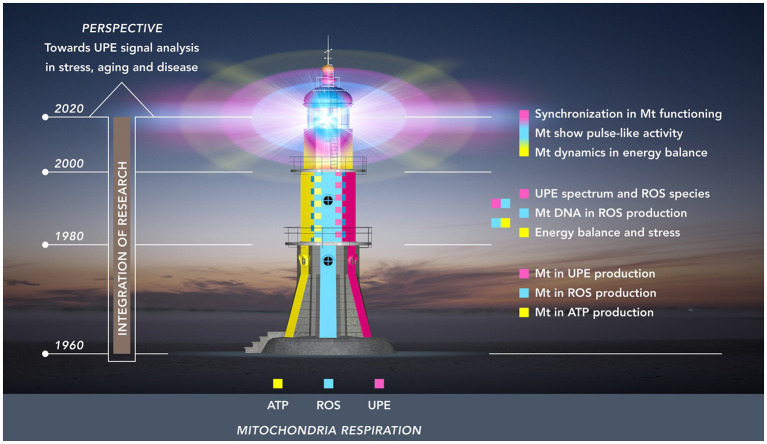
Research towards the mitochondrial functioning as a “(ultra-weak) light house” organelle. The light house from base till top represents three periods of 20 years: 1960–1980 (lowest floor); 1980–2000 (mid-floor); 2000–2020 (top-floor). The colors illustrate three developing fields of research related to mitochondria: energy (ATP) production (yellow); ROS production (cyan blue); UPE production (magenta red). These research fields originally developed independently in the first period. In the second period, two integrations took place: (a) energy balance and ROS production and (b) ROS production and UPE. The third period (top floor) shows the intertwining of colors representing the dynamic pulsation kinetics in energy and ROS production and the development of procedures to detect analogous pulsation in UPE signals of living tissue.

To conceptualize the timeline of this integration and, at the same time, emphasizing a future role of UPE, we used a lighthouse as a metaphor (Figure 1). In this depiction, the first period (1960–1980) is represented by the lower layer: a formative period during which each of the three autonomous research areas acquired its own independent body of empirical knowledge and methodological expertise. These largely unconnected knowledge systems served as the building blocks that would later – in the period between 1980 and 2000 – be integrated in the second pile of the lighthouse structure. To begin with this integration, this period saw the booming of research that focused on the critical role of mtDNA for mitochondrial functioning, and on the specific sensitivity of mtDNA for ROS – this research resulted in the vicious cycle theory of mitochondrial ROS production, and demonstrated the role of ROS in increasing the probability of damage and stress-related and age-related metabolic and degenerative diseases. Also, during this period, integration was further enhanced by progress made in the investigation of the relationship between ROS and UPE in stress and aging.

The third and final integration – which is still going on – is depicted in the dome of the mitochondrial lighthouse with its major characteristic of dynamic signaling; as such, it represents the most recent technological advances in mitochondrial research (2000–2020) on dynamics in mitochondrial structure and the flash-like production of ROS, and a first step moving to organ levels with synchronized flash patterns. Extrapolating from this situation at microscopic scale level, the authors will review some research at macroscopic level on how the non-invasive recording of UPE and its analysis may contribute to our understanding of health and disease.

## Three Research Areas and a Building Layer (1960–1980)

In section Three Research Areas and a Building Layer (1960–1980), our overview will discuss mitochondria-related research in the period between 1960 and 1980, a period in which research focused on three areas that were thought to be largely independent at the time: respiration and energy production, the production of reactive oxygen species, and ultra-weak photon emission (Figure 1, lowest floor). During this formative period, each of the three research areas acquired an independent body of knowledge, thereby providing the building blocks that would later be integrated.

### Mitochondria Are Powerhouses

Mitochondrial oxidation was confirmed in 1949, when Kennedy and Lehninger demonstrated that the mitochondrion (identified as the site of the cellular respiratory assembly) contained the enzymes of the citric acid cycle (Kennedy and Lehninger, 1949). A typical mitochondrion is about the size of a bacterial cell with a diameter of 0.2–0.8 μm and a length of 0.5–2 μm. Published in the early 1950s, the first high-resolution electron micrographs of mitochondria showed their two bounding membrane systems (Palade, 1952; Sjöstrand, 1953). Both systems were later found to have markedly different biochemical and functional properties.

Considering these properties, it was found that the outer mitochondrial membrane has few proteins, with the major protein forming channels through this membrane that allow a free diffusion of ions and small water-soluble metabolites. In contrast, the inner mitochondrial membrane was shown to be rich in protein. This inner membrane is folded into a series of internal ridges called “cristae” and forms a barrier to protons and larger polar and ionic substances, which may cross this membrane only *via* specific transporters. The two membranes create two submitochondrial compartments: (1) the intermembrane space, i.e., the region between the inner and outer membranes and (2) the matrix, which is bounded by the inner membrane.

The reactions of the citric acid cycle take place in the matrix, which also contains a pool of NAD^+^ and NADP^+^ that remains separate from pyridine nucleotide enzymes of the cytosol. Oxidative phosphorylation (OXPHOS) takes place in the inner mitochondrial membrane. This cellular respiration starts with the formed strong reducing agents NADH and FADH_2_ and is the culmination of a series of oxidation-reduction transformations that utilize the four enzyme complexes (I–IV) of the mitochondrial respiration chain. The flow of electrons from NADH or FADH_2_ to O_2_ through these protein complexes leads to the pumping of protons out of the mitochondrial matrix. The resulting uneven distribution of protons generates a pH gradient and a transmembrane electrical potential that creates a proton motive force. Finally, these protons flow through ATP synthase, the enzyme that catalyzes the synthesis of ATP from ADP and inorganic phosphate (Mitchell, 1961).

### Respiration as Major Producer of Reactive Oxygen Species

In a 1954 study by Gerschman and Gilbert, it was proposed that most of the damaging effects of elevated oxygen concentrations in living systems could be attributed to the formation of free oxygen radicals (Gerschman et al., 1954; Gerschman, 1981). As a further elaboration of this idea, Harman postulated his free radical theory of aging, a theory that regarded the accumulating and irreversible damage to cellular macromolecules caused by oxygen radicals as the single common factor in all aging processes (Harman, 1956). However, in the mid-1950s, it was felt that free radicals were far too reactive to exist in any great quantity in biological materials, and ROS-related hypotheses did not capture much interest until 1969, when free radicals were further implicated by the discovery of the enzyme superoxide dismutase (SOD) (McCord and Fridovich, 1969). SOD was found to catalytically remove superoxide anion (O_2_^−^), the primary ROS in living systems, a free radical known to generate secondary substances that are highly reactive (Chance et al., 1975; Kellogg and Fridovitch, 1975). Still, many researchers remained unfamiliar with free radicals, and the field was generally regarded as irrelevant to mainstream biology until the 1980s, when evidence was found that reactive oxygen species actually produce alterations in DNA, proteins, and lipids (Halliwell, 2007).

In understanding the central role of molecular oxygen in the production of ROS, it was noted that molecular oxygen itself is actually a free biradical (Jamieson, 1989). The single oxygen atom is unstable and tends to bind a twin atom; in the molecular oxygen that results, one pair of electrons is shared, and two unpaired electrons remain. With no twin atom present, a single oxygen atom may accept hydrogen to form water (H_2_O). In this case, a full reduction of O_2_ requires the transfer of four electrons, which provide for two molecules of H_2_O. Such a complete strategy for the full reduction of O_2_ is accomplished *vis-à-vis* the cytochrome c oxidase complex, since this complex does not release partly reduced intermediates by holding O_2_ tightly between Fe and Cu ions. The absence of this protection will result in a partial reduction of O_2_.

In living systems, superoxide anion (O_2_^−^) is created from oxygen by electron addition within the mitochondrial respiratory chain (Taylor et al., 1986; Halliwell, 1991). As mentioned before, superoxide anion is a free radical that is able to generate secondary substances that are highly reactive and may interact with other molecules, thereby giving rise to chain reactions. The chain reactions following superoxide anion production include both oxygen radicals and certain non-radicals that are easily converted into radicals (singlet oxygen: ^1^O_2_ and hydrogen peroxide: H_2_O_2_). The collective term for this group of both radical and not-yet-radicalized oxidizing agents is ROS – in a “bio-logical” sense, therefore, all oxygen radical species belong to the ROS category, though conversely, not all ROS are oxygen radicals (Halliwell, 2007). With oxygen-centered, nitrogen-centered, carbon-centered, and sulfur-centered radicals, we nowadays differentiate between a number of categories of free radicals – nonetheless, in living systems, oxygen radicals still remain the most important class of radical species (Buonocore et al., 2010).

### Respiration as Major Producer of Ultra-Weak Photon Emission

The first documented reports of radiation emanating from living organisms were published in Italy and, in the early 1960s, in Russia (Colli and Facchini, 1954; Colli et al., 1955; Popov and Tarusov, 1963; Veselowskii et al., 1963; Tarusov and Zhuravlev, 1965). In these studies, sensitive photomultiplier tubes were utilized to study photon emission from organisms and tissues. Since then, further advances in photodetection with extremely sensitive photomultiplier tube technology have confirmed that many, if not all, living systems emit very low levels of UV, VIS and NIR photons (Van Wijk, 2014). Originally described as spontaneous ultra-weak chemiluminescence, this biological emission was later renamed to spontaneous UPE. Early research found that the intensity of photon emission depends on the concentration of oxygen in the atmosphere. Next, in search of the biological compounds that actually produced the chemiluminescence, carefully controlled tissue homogenization procedures were carried out that could break up tissues but allowed cell organelles to remain intact. These procedures facilitated the identification of the mitochondria as the cellular parts that were responsible for the majority of the photon emission (Tarusov and Zhuravlev, 1965). Moreover, it was demonstrated that the luminescence of mitochondria is associated with oxidative phosphorylation (Vladimirov and L’vova, 1964). These classic research data were first reviewed in Barenboim et al. (1969).

From the early 1970s onwards and following the Russian example, a number of research laboratories began to utilize photomultiplier equipment in the study of ultra-weak photon emission from biological organisms. Soon, experimental data were provided by research groups in Japan, Australia, and Poland, followed shortly by teams in Germany, United States, and China. Interestingly, these research groups were motivated by different backgrounds. Most teams studied the biological photon emission from the biochemical point of view, asking questions about its biochemical background. Two research groups had a different interest. The German approach focused on a possible regulatory role of photons within cells utilizing a biophysical approach based on Gurwitsch’ mitogenetic radiation studies (Volodyaev and Beloussov, 2015). The Chinese approach focused on a possible role of photons in energy concepts of traditional Chinese medicine. As a recent overview shows, by 1980 the proliferation of research had resulted in a world-wide exploration of ultra-weak photon emission phenomena (Van Wijk, 2014), part of it, namely the biochemical oriented studies, are used in this review to illustrate the possible role of ultra-weak photon emission in mitochondrial research.

In this first section, we have outlined the three independent areas that shaped mitochondria-related research between 1960 and 1980. In the next section, as presented in the mid-floor of the light house in Figure 1, we will learn how mitochondrial research progressed during the 1980s and 1990s, and how two types of integration have taken place: on one hand, the role of ROS in mitochondrial functioning in stress and aging, and, on the other hand and independently from the first integration, the role of ultra-weak photon emission in research that focused on ROS-production in stress and stress adaptation.

## A Second Layer: The First Period of Integration (1980–2000)

Between 1980 and 2000, mitochondria-related research saw a first period of mutual integration of the “autonomous” research areas that were outlined in the previous section. This partial integration took shape in the merging of empirical findings and in a combining of methodological expertise, both in the role of ROS in mitochondrial functioning in stress and aging (section ROS in Mitochondrial Functioning and Aging), and in the role of UPE in research on ROS and stress (section Connecting Ultra-Weak Photon Emission, ROS, and Stress).

### ROS in Mitochondrial Functioning and Aging

It was only when mitochondrial DNA research started to boom in the 1980s the integration of ROS and mitochondrial functioning started. At that time, the enormous complexity of mitochondrial functioning became clear as well as the dual principle of anterograde and retrograde signaling between mitochondria and nucleus. Since the early 1960s, it was known that mitochondria house their own DNA: mtDNA (the mtDNA is considered a vestige of bacterial heritage). Present in each human mitochondrion, mtDNA consists of one or more small (16.6 kb) double stranded circles of DNA. With the entire human mtDNA sequence determined in Anderson et al. (1981), a number of genes critical for mitochondrial functioning were identified. These included the genetic coding for 13 respiratory chain polypeptides and for the 24 nucleic acids (two ribosomal RNAs and 22 transfer RNAs) that are needed for intramitochondrial protein synthesis. All mtDNA-encoded proteins are subunits of the respiratory chain complexes I, III, IV, and V. Data also revealed that mitochondrial proteins were encoded by both mtDNA and nuclear DNA (nDNA). Nuclear genes code for the majority of mitochondrial respiratory chain polypeptides; also, complex II (succinate dehydrogenase) is fully encoded by the nuclear genome.

Not only is mtDNA less complex, it is also much less protected than the coiled and chromatin-packaged nuclear DNA. The mutation rate of mtDNA is 10–20 times higher than that of nuclear DNA, and mutagens bind to mtDNA up to a 1,000 times stronger, all of which leaves mtDNA far more susceptible to damage in comparison to nuclear DNA. Also, DNA repair mechanisms are much less efficient in mitochondria. These observations imply that mtDNA damage and mutation occur relatively easy, while at some point the subsequential impairment of respiration will begin to impact on mitochondrial functioning in meeting the cell’s energy demands. Combined with data on the relatively high production of ROS in mitochondria (section Respiration as Major Producer of Ultra-Weak Photon Emission), this vulnerability suggested a high chance of mtDNA damage stemming from ROS (Fleming et al., 1982, 1985; Marcus et al., 1982; Miquel and Fleming, 1986).

To explain the high ROS production in mitochondria, the vicious cycle theory of mitochondrial ROS production states that accumulating levels of ROS in mitochondria are produced by reactions of ROS species with the mitochondrial membranes, a process that results in chain reactions of lipid peroxidation – in these chain reactions, lipid peroxyl radicals formed at a previous stage can react with oxygen, thus restarting the cycle of radical production (Halliwell and Gutteridge, 1985; Halliwell, 1991). However, at first instance, more ROS production and subsequent more mtDNA damage has not an immediate impact on cell’s energy demands. To fully understand the impact of ROS production on mtDNA damage and the energy demand of the cell, we first have to take into account the large number of mitochondria and mtDNA copies per cell, which, depending on the cell type, ranges from 1,000 up to 100,000, with all copies being identical in a healthy individual at birth. Obviously, then, a single mtDNA mutation or deletion would not automatically lead to a damaged or diseased cell state; in fact, the mitochondria of patients harboring pathogenic mtDNA defects often show a mixture of mutated and non-mutated (wild-type) mtDNA, a mixture in which the percentage of mutated mtDNA can vary widely from organ to organ, and even between individual cells within the same organ. It therefore became clear that, in addition to the vicious cycle theory of mitochondrial ROS production, the time parameter had to be considered an important criterion in our understanding of the effects of mtDNA damage in cell functioning. Accumulating in time, oxidative mtDNA damage constitutes a “normal aging process,” an apparently irreversible process that determines a risk factor for the development of age-associated disorders, such as cancer and neurodegenerative diseases.

In addition to this time-related damage to mtDNA in the long-term, the increasing levels of ROS potentially may cause indirect “secondary” damage by a gradually increasing source of secondary products of lipid peroxidation, such as mutagenetic aldehydes, malondialdehyde (MDA), and 4-hydroxynonenal/4-hydroxy-2-nonenal (HNE), products that are considered markers of oxidative stress. They have unique properties compared to ROS itself, in that the non-charged structure of aldehydes allows them to easily migrate through membrane and cytosol, which, consequently, enables them to cause far-reaching damaging effects inside or outside the cells. For example, HNE and MDA not only modify the amino acid residues leading to protein damage, they may also form covalent adducts with nuclear DNA and mtDNA damage of various types: double and single-strand breaks, intra‐ and inter-strand DNA crosslinks, DNA adduct formation, and DNA modifications.

To further add to our understanding of the long-term effects of mitochondrial malfunctioning on nuclear gene expression, we have to consider other types of pathways in the functional interaction between mitochondria and the cell’s nucleus. This dual functional interaction becomes clear, if we differentiate between the anterograde and the retrograde signals. Anterograde signals are defined as the signals directed from the cell nucleus to the mitochondria. In accordance with the energy and growth requirements of the cell, such signals control the import of proteins in mitochondria and the availability of substrates, and they regulate the maintenance of mtDNA and the expression of its genes. Retrograde feedback on the other hand, is defined as a type of signal directed from mitochondria to nucleus. In a healthy cell, under normal physiological conditions, this retrograde feedback is likely to consist of the normal mitochondrial output of ATP, metabolic intermediates, and basal reactive oxygen radicals. However, in pathogenic mtDNA with mitochondria under stress, this retrograde mitochondrial signaling has the ability to bring about global changes in the nuclear gene expression and phenotype in cells.

One type of retrograde signal pathway involves “metabolic epigenetics,” a concept that refers to the changes in mitochondrial output that result in alterations of chromatin and other factors that regulate nuclear gene expression. It is through this pathway, that mitochondria provide key metabolites [e.g., beta-nicotinamide adenine dinucleotide (NAD^+^), ATP, alpha-ketoglutarate, and acetyl coenzyme A] that are co-substrates required for numerous transcription regulatory processes. These metabolites mediate gene expression changes that control cellular processes *via* chromatin structure changes and dynamics, DNA methylation, histone modification, and noncoding RNA expression. Epigenetic modifiers include DNA methyltransferases, histone acetyltransferases, histone deacetylases, sirtuins (SIRTs), histone demethylases, poly (ADP-ribose) polymerase, and other enzymes that work coordinately in regulating gene expression.

Another type of pathway in retrograde signaling is initiated by unfolded proteins. Mitochondrial stress disturbs the mitochondrial membrane structure and exposes proteins to a continuous source of danger affecting protein homeostasis in the matrix, in which oxidatively damaged proteins and unfolded proteins are degraded by mitochondrial proteases. Quality control of mitochondrial functioning requires that the unfolded proteins signal is communicated from the mitochondrial matrix to the cell nucleus, in order to induce the appropriate nuclear DNA response to this type of toxic stress. From the early 1980s onward, recognition of the importance of a response to stress “from within” the cell had moved the molecular stress response to center stage in cell and molecular biology (Schlesinger et al., 1982). Those studies are related to the strain caused by macromolecular structures that have been rendered useless by damage, i.e., structures that need the attention of either repair or decay systems, since they obstruct the normal functioning of the cell. This stress response will involve the gene expression and synthesis of a special group of proteins: heat shock proteins (HSP) or stress proteins (Schamhart et al., 1984; Van Wijk and Schamhart, 1988; Wiegant et al., 1993; Ovelgönne et al., 1995a), a type of proteins that function as “chaperones” (a term first proposed by Ellis and Van Der Vies, 1988) in that they prevent incorrect interactions with other proteins and assist unfolded proteins in their correct refolding (Ellis et al., 1989; Pardue et al., 1989). A number of studies were conducted that focused on the mechanism of ROS-induced expression of genes encoding for stress proteins (Burdon et al., 1990; Ovelgönne et al., 1995b), while other research started to focus on the role of heat shock proteins in tolerance development (Schamhart et al., 1984; Van Dongen et al., 1986) and their functioning as chaperones (and chaperonins) in protein folding and trafficking, among others to the mitochondria (Welch et al., 1989).

Pathogenic mtDNA mutations associated with human disease were first discovered in (Holt et al., 1988; Wallace et al., 1988). It was a breakthrough discovery that paved the way confirming that not only mtDNA mutations but also specific alterations of nuclear gene expression were caused by responses to mitochondrial dysfunctioning. As the mtDNA codes for the central genes of the mitochondrial energy-generating process of OXPHOS, the diseases that may result from mtDNA mutations are usually metabolic and degenerative in nature. Milder variants of mtDNA mutation may affect caloric metabolism. These mutations may result in metabolic abnormalities such as diabetes and obesity, but they may also affect energy-demanding organs such as the brain, thereby leading to late-onset degenerative diseases (e.g., Parkinson’s disease and Alzheimer’s disease). Relatively more severe mtDNA mutations, such as myoclonic epilepsy with ragged fibers syndrome (MERRF) and mitochondrial encephalopathy, lactic acidosis, and stroke-like episodes syndrome (MELAS), cause progressive multisystem diseases, frequently resulting in premature death. The most severe mtDNA mutations can lead to lethal childhood diseases, such as Leigh syndrome (also called subacute necrotizing encephalomyelopathy). The growing list of defects has pushed mitochondriopathies into the realm of many medical specialties, including endocrinology, gastroenterology, immunology, neurology, oncology, and others (Chinnery, 1993; Schon et al., 1997; Wallace, 1999).

At the end of the first integration in the period 1980–2000, it was evident that progression of ROS-related damage is important to understand the phenomenon of stress, aging, and aging-related diseases. In this type of research, no attention was paid by these researchers to the research field of ultra-weak photon emission. However, the latter field became interesting because in the same period 1980–2000, ultra-weak photon emission was definitively linked to ROS and stress.

### Connecting Ultra-Weak Photon Emission, ROS, and Stress

In the scientific community that focused on UPE of living organisms, it was commonly accepted that almost all living organisms produce this ultra-weak light. In contrast, there was a definite need to explain fluctuations in UPE strength to understand the origin of UPE, both physiologically and biochemically. To address these questions, a multi-author review was published in 1992 under the title “Biophoton emission, stress and diseases” (Van Wijk et al., 1992). This review covered three decades of research by teams that had improved their technology and were able to study and characterize UPE in relation to ROS and perturbation of organisms. The studies had included bacteria (Tilbury, 1992), yeast (Slawinski et al., 1992; Tilbury, 1992), amoeba (Fisher and Rosenberg, 1988; Fisher et al., 1991; Tilbury, 1992), plants (Ruth and Popp, 1976; Slawinski et al., 1992), and animals (Boveris et al., 1980, 1981; Cadenas et al., 1980a,b; Inaba, 1988). In these studies, organisms had been exposed to temperature, oxidative, metabolic, chemical, or photochemical (UV) induced perturbations; in most instances, the perturbation resulting from these stimuli had led to an increase in photon emission. Below, we present first some classic studies on increased UPE in stress; they are followed by more advanced studies including UPE and ROS in stress-exposed organisms.

In the case of plants, studies on the relation between temperature stress and photon emission of grain seedlings demonstrated that life-threatening temperature conditions were associated with increased emissions. The UPE intensity of seedlings increased dramatically both at an “upper temperature growth limit” and at a “low temperature growth limit.” Both limits determined life-threatening conditions for the seedlings. Both limits represent severe stress conditions that result in death of the seedling and which are paralleled by increased UPE. It was already known that selection of seeds has resulted in grain species with different resistance to temperature, showing altered upper or low temperature growth limits. The study of UPE of such variants cultivated at different temperatures has demonstrated that the upper and lower levels of UPE increase of different grain species correlated with, respectively, their heat resistance and frost resistance (Tarusov and Veselovski, 1978).

An example of a more advanced approach has used yeast cultures, a less complicated experimental model system, to explore the connection between UPE, ROS, and stress in metabolic adaptation. In yeast, UPE was studied during growth of oxygenated cultures on glucose-rich media from their exponential state into the stationary state. This growth requires a metabolic shift when glucose becomes limiting, a phenomenon that is known as “glucose effect” or “catabolite repression” (Polakis et al., 1964; Polakis and Bartley, 1965; Van Wijk et al., 1969). Yeast cells start to grow preferentially with glucose as substrate even if other metabolic substrates are available; glucose strongly repressing the formation of mitochondrial structures and the synthesis of respiratory enzymes and trisaccharide or disaccharide splitting enzymes. Although oxygen is available, glucose is metabolized *via* glycolytic fermentation and only when glucose has been almost consumed, the yeast cells adapt to other carbon sources available in the medium or to the alcohol formed from glucose by glycolysis and excreted in the growth medium. The metabolic adaptation consists of the synthesis of respiratory enzymes and the development of mitochondria allowing cells to use alcohol for the next growth phase. In this phase, oxygen is absolutely required for respiration. This yeast model system has demonstrated increased UPE during the metabolic adaptation (Quickenden and Tilbury, 1983). The novel step in this second example was the introduction of spectral analysis in analyzing UPE of a biological model to investigate the nature of the chemical reactions that are responsible for the increased photon emission in metabolic adaptation.

The inclusion of spectral analysis procedures in biological research was based on knowledge about the nature of the biochemical reactions that are responsible for the increased photon emission. By the mid-1980s, these state-of-the-art procedures were the central topic of several reviews (Slawinska and Slawinski, 1983, 1985; Cadenas, 1984; Inaba, 1988; Popp et al., 1988; Slawinski, 1990, 1991). By that time, the combination of super-high sensitivity photon-counting systems and advanced spectral analysis techniques and procedures had led to the successful identification of various ultra-weak photon emission bands in the range of 400–800 nm (Slawinska and Slawinski, 1983, 1985; Cadenas, 1984; Cadenas et al., 1984; Inaba, 1988). This data then served as much needed indications of the biochemical reactions that produce UPE in living organisms. Regarding the early application of these findings to experimental stress research, it is important to note that the reliability of the data depended on the accuracy of the specific spectral analysis technology involved. In this new research area, studies showed UPE to be brighter during the metabolic transition (Tilbury, 1990, 1992), while spectrum analysis revealed that this brighter emission occurred mainly in the spectral band from 450 to 600 nm. The studies were performed with three yeast species: *Saccharomyces cerevisiae*, *Saccharomyces pombe*, and *Candida utilis*. For each of the three species of yeast examined the exponential phase emission comprised both a UV (210–330 nm) band and a visible region band (450–620 nm), the latter band was about 2.5 times brighter than the first band. After adaptation, in the stationary growth phase, the visible band was specifically increased up to seven times its previous value. A similar spectral increase was detected when yeast cell respiration was challenged by growing the cells in the presence of appropriate dyes, such as acriflavine, resulting in mtDNA damage, increased ROS production and corresponding respiratory deficiency. In such conditions, the UPE peak was about 10 times as intense as normal. Spectral analysis studies showed that the observed brightness was dominated by a band around 600 nm (Quickenden and Tilbury, 1983, 1991), which served as an indication that lipid peroxidation reactions were involved in UPE. Nevertheless, these studies failed to present more detailed conclusions about the ROS species involved due to the precision limits of the technology used, which only resolved the spectral area of interest (an area ranging from 210 to 630 nm) in seven areas with a broadness of roughly 70 nm each.

The broad range of spectral bands of photon emission corresponding with production of ROS species became evident when most common cellular reactions were mapped. To summarize this map, we will start with superoxide anions (O_2_^−^) (section Respiration as Major Producer of Reactive Oxygen Species), which may appear on both sides of the inner mitochondrial membrane, and as such, they are also located in the matrix space. Within both compartments, enzymes are present that may specifically react with superoxide anion. These are two different SOD enzymes, a manganese containing enzyme (located at the matrix side) and a copper zinc containing enzyme (located at the intermembrane space). Both SODs transform superoxide anions (*vis-à-vis* protons) to hydrogen peroxide and excited oxygen in the triplet state (^3^O_2_^✽^). This excited, high energy state of O_2_ is able to emit a red photon in the transition to O_2_. Hydrogen peroxide molecules proved to be another source of radiation when they spontaneously dismutate to water and excited singlet oxygen (^1^O_2_^✽^), which (upon decay to the ground state) may also emit a red photon. Excited singlet oxygen molecules can emit in the 780 nm or in bands that lie even further into the IR. Other emission bands, centered on 634 and 703 nm, arise after dimerization of excited singlet oxygen. The excited singlet oxygen molecules may excite a secondary emission by reacting with lipids (especially unsaturated fatty acids), in which the methylene groups in double bonds possess reactive hydrogen atoms. By the dismutation reactions of peroxy radicals and the cleavage of peroxides, the most common emitters of UPE are produced: carbonyl compounds (>C=O) in the excited singlet and triplet states, as well as excited dimers (dimols) of the singlet molecular oxygen ^1^O_2_^✽^. Excited carbonyl groups may be responsible for the emissions at wavelengths within the 350–500 nm range. Upon incomplete reduction of H_2_O_2_, the hydroxyl radical (OH^•^) is generated (known to be one of the strongest oxidants in nature). The hydroxyl radical may also lead to the emission of photons when it reacts with lipids, thus resulting in the formation of excited carbonyl species (ROO^•^), in which oxygen is in the excited singlet or triplet states. Upon decay to the ground state, these carbonyl species emit photons. It should also be emphasized that recombination reactions of certain radicals may release up to 480 kJ/mol energy, which is sufficient to generate UV-photons in the 230–300 nm range. This summary of the range of spectral bands associated with ROS-related reactions suggests that detailed conclusions need more spectral areas of interest, and hence a broadness of bands for analysis of less than 70 nm.

A more sophisticated spectral analyzer system for ultra-weak intensities was then developed with high optical efficiency to minimize the optical loss (Inaba, 1988). It had the following main features: the entrance aperture was large and independent of the resolving power and a set of colored glass filters was employed as a wavelength selector, which possesses different sharp short-wavelength cutoffs instead of a combination of a wavelength dispersion element, such as a grating or a prism, and a slit. It was confirmed that the glass filter generally has a wider acceptance angle than the interference filter without appreciable shifts of the transmission wavelengths. Also, the transmission of a colored glass filter was usually much better than that of an interference filter and a wavelength dispersion element. The more sophisticated spectral analysis of ultra-weak light signals was accomplished with the successive insertion of colored glass filters to cover the total wavelength region between 275 and 670 nm by 27 colored filters with individual spectral windows of 30–50 nm for the wavelength region 275–450 and 20–25 nm for the wavelength region 450–670 nm.

Other researchers have estimated the spectral analysis of UPE of yeast utilizing 11 spectral areas with a broadness of roughly 20–25 nm within the 470–700 nm range plus three spectral areas within the 670–830 nm range (Ezzahir et al., 1992; Slawinski et al., 1992). They exposed yeast (*S. cerevisiae*) to a formaldehyde stress which leads to intra‐ and inter-molecular cross-linking by ‒CH_2_‒ bridges and subsequent denaturation of proteins (Slawinski, 1990, 1991; Ezzahir et al., 1992). In these studies, the intensity of UPE increased with the increase of the concentration of formaldehyde in the range 0.06–7% by a factor up to 70, while oxygen consumption by yeast cells decreased. The spontaneous UPE of native yeast cells covered predominantly the red region above 700 nm. In the presence of formaldehyde, a blue-green transient emission appeared (with peaks at 540 and 630 nm), then it weakened with time and a strong emission in the red region with a maximum of 620–660 nm appeared.

With these examples, it was evident that non-invasive recording of shifts in ROS species in stress has become possible for laboratories possessing the advanced technology for spectral analysis combined with highly sensitive photomultipliers. Unfortunately, this technology has remained limited to only a few laboratories. Nevertheless, the examples do show that this technology offers sufficient promise to non-invasively record mitochondrial functioning *vis-à-vis* their ROS production in cultured cells. Progress in further integration of the research presented in section ROS in Mitochondrial Functioning and Aging and section Connecting Ultra-Weak Photon Emission, ROS, and stress has been seriously hampered, mainly for two reasons. Firstly, the few institutes with facilities for ultra-weak photon emission studies were mostly interested in biophysical aspects of photon emission and not familiar with mitochondrial biology and progress in mitochondrial medicine. Secondly, major attention in mitochondrial research focused in a novel direction including a more detailed insight into regulatory processes as will be discussed in section Enhanced Integration: Mitochondrial Dynamics (2000–2020). At the end of that section we will return again to the possible contribution of ultra-weak photon emission to these novel developments.

## Enhanced Integration: Mitochondrial Dynamics (2000–2020)

Whereas in the previous period of mitochondrial research, the incomplete merging of research areas was characterized by two-sided interrelations (Sections ROS in Mitochondrial Functioning and Aging and Connecting Ultra-Weak Photon Emission, ROS, and Stress), a gradually more developed overall integration took place during the next period in our review (2000–2020). This third level of integration, as depicted in the top floor of the lighthouse in Figure 1, saw the emergence of some new dynamics in mitochondria: mitochondrial structural dynamics, mitochondrial ROS production dynamics, and novel characteristics in the UPE signal.

### Mitochondrial Structural Dynamics

Although textbooks traditionally depict mitochondria as static organelles, by the early 2000s, it had become evident that mitochondria do not sit idle within the cell. The introduction of deconvolution microscopy (i.e., confocal scanning tools for the three-dimensional visualization of live cells) had revealed that the mitochondrial network undergoes extensive remodeling in a constant dynamic process. In this process, mitochondria may change their shape as the result of both fission (dividing in separate parts) and fusion (leading to an increase in size) as reviewed by Liesa et al. (2009).

Since then, the molecular biology of mitochondrial movement, tethering, fusion, and fission events has been extensively studied. A number of proteins that mediated fission were identified: dynamin-related protein 1 (Drp 1, consists mostly of cytosolic and translocates to the outer mitochondrial membrane during fission), fission 1 protein (Fis 1, located in the outer mitochondrial membrane), and mitochondrial fission factor (Mff, also located in the outer mitochondrial membrane; Frank et al., 2001; Lee et al., 2004). In fact, Mff and Fis 1 mediate fission by recruiting Drp 1 to the mitochondria, which requires guanosine triphosphate (GTP) hydrolysis. On the other hand, mitochondrial fusion is mediated by the optic atrophy gene 1 (Opa 1, located in the inner membrane) and mitofusins (Mfn1 and Mfn2, located in the outer mitochondrial membrane) (Lee et al., 2004; Ishihara et al., 2006). These three proteins require GTPase activity in mediating fusion and remodeling the internal cristae structure of the inner membrane.

The fusion and fission machinery is a vital part of the continuing mitochondrial life cycle that enables the reorganization of mitochondrial components necessary to optimalize bioenergetic functioning of the mitochondrial population within the cell (Twig et al., 2008a; Molina et al., 2009; Mouli et al., 2009). Studies of mitochondrial dynamics have identified an intriguing link between the mitochondrial architecture and the energy supply (Benard et al., 2007). Cells exposed to rich nutrient environments tend to keep their mitochondria in a fragmented state, while mitochondria in cells under starvation were shown to prolong their connected state. These observations suggest that the functionality of the shift in the fusion-fission balance is related to damage and repair. In metabolic rich environments that are characterized by excessive ROS generation and molecular damage, the formation of inefficient ATP producing parts of mitochondria may be avoided through the process of fission. Thus, fission causes a dilution of these inefficient parts in separate regions of the network, hence facilitating an optimal repair of defective mitochondria. Moreover, fission may also isolate dysfunctional mitochondrial parts and thereby facilitate directing these parts along the autophagy route, thus avoiding the accumulation of damaged mitochondria (Parone et al., 2008). On the other hand, the fusion process dilutes damaged molecules and may protect functioning mitochondria from engulfment with autophagosomes (Twig et al., 2008b).

### Mitochondrial ROS Production Dynamics

Additional details of this structural dynamics were observed utilizing the confocal microscopic technique for the visualization of single mitochondria, tracked over time using specific mitochondrial function sensitive fluorescent substances. The application of novel fluorescent tools to probe for the activity and substances confined within a single individual mitochondrion was a recent breakthrough. These tools enable the study of mitochondrial functioning over time in that they measure a variety of intra-mitochondrion processes, such as the formation of superoxide, mitochondrial Ca^2+^ uptake and mitochondrial membrane potential, and pH.

Utilizing these advanced instruments, Wang analyzed the mitochondrial formation of superoxide (considered the primary ROS) by applying a mitochondrial-targeted biosensor cpYFP protein (Wang et al., 2008). Surprisingly, his results revealed acute increases in fluorescence within the mitochondrial matrix, a truly remarkable phenomenon, since superoxide formation had previously been thought of as a steady-state level process. The pulsed mitochondrial superoxide formation proved to be a transient and stochastic, “flash”-like event – the persistence of fluorescent flash activity being over a period of 10–15 s. The flashes in fluorescence have been detected in the mitochondria of a range of mammalian tissues and cells (Li et al., 2012; Wang et al., 2012, 2013), and though the frequency of flash events differed across the different cell types, the kinetic properties and durations of individual flash events were observed to be essentially invariant. Moreover, while superoxide within the mitochondrial matrix is rapidly dismutated to hydrogen peroxide, the cpYFP protein biosensor is not sensitive to the resulting hydrogen peroxide (Wang et al., 2008), which is consistent with flashes representing a robust burst of superoxide production per se.

A related breakthrough discovery involved the development of fluorescent substances for recording other processes related to mitochondrial respiration, such as Ca^2+^ uptake, pH, and membrane potential. Probing these mitochondrial processes showed the same kinetic features as were detected with cpYFP (Wei and Dirkson, 2012; Wang et al., 2016); moreover, synchronized flashing was recorded in studies that monitored two or more mitochondrial targeted fluorescent indicators simultaneously, illustrating that a single flash event involves multiple transient concurrent changes within the mitochondrion, including changes in ROS generation, Ca^2+^ homeostasis, membrane potential, and pH. Taken together, these observations supported the notion that superoxide signaling – and, presumably, the associated ROS-related UPE – should be considered an on-off phenomenon, like all respiration-related processes in the mitochondrion sharing the common feature of a strong dependence on the electron transport chain (Wang et al., 2008; Azarias and Chatton, 2011; Schwarzlander et al., 2012; Santo-Domingo et al., 2013; Wei-LaPierre et al., 2013; Breckwoldt et al., 2014; Gong et al., 2015). In line with that, several studies have reported that mitochondrial flashes in living cells, tissues, and animals are stimulated by physiological respiration substrates, including oxygen, glucose, fatty acids, and specific substrates for the electron transport chain complexes (Wang et al., 2008; Pouvreau, 2010; Fang et al., 2011; Wei et al., 2011a,b; Santo-Domingo et al., 2013; Gong et al., 2014, 2015; Shen et al., 2014).

If we return to the visualization of the dynamics of mitochondrial structure, it is of interest that, using this procedure, Wikstrom et al. (2007, 2009) discovered that a fission event may generate two bioenergetically different mitochondria, one with a higher membrane potential and one with lower membrane potential. This is of interest for better understanding the role of mitochondrial life cycle in dysfunctional mitochondria. Hence, the daughter mitochondrion with lower membrane potential is faced with two future options: either it recovers its membrane potential and regains its fusion capacity to reconnect with the network, or it will be left depolarized. The latter perpetuation of their solitary state renders such mitochondria dysfunctional; as such, they comprise the pre-autophagic pool of mitochondria to be degraded (Twig et al., 2010).

Mitochondrial flash events reflect a fundamental physiological phenomenon that has a significant functional impact on the type of regulation in bioenergetic adaptation at mitochondrial level. This type of regulation offers mitochondria the possibility to adjust for an increased or decreased energy demand of a cell by changing the frequency and number of its metabolic pulses of ATP production. A further discovery along this line is the characterization of mitochondrial superoxide flash properties in skeletal muscle regarding the spatial dynamics, demonstrating that in a certain space mitochondria are not flashing randomly but in a synchronized manner (Pouvreau, 2010; Wei et al., 2011a,b). The studies utilized automated detection and analysis program for the identification and quantitation of flash frequency, amplitude, full duration at half maximum, time constant of decay, and spatial area of superoxide flashes during the time series. These recordings of flash activity in skeletal muscle fibers demonstrated that individual mitochondrial events exhibit a variety of spatial morphological appearances, ranging from synchronous single flashing mitochondria amid many non-flashing ones, to synchronous flashing events in multiple adjacent mitochondria, which were longitudinally or transversally arranged surrounded by quiescent ones, to appearances of flash events encompassing large clusters of mitochondria. Even the larger cluster-sized flashes presented a striking homogeneity in terms of increase of fluorescence intensity. Furthermore, the same cluster could flash several times during a 3 min record, the shape of the successive flashes being identical. Overall, results show the existence of intermyofibrillar mitochondrial units of variable size, characterized by the synchronization of their superoxide production. Similar observations have been confirmed in the case of the heart muscle (Wang et al., 2012).

To conclude this section, it can be stated that mitochondrial superoxide flash activity may reflect the synchronized activity of individual mitochondria (Aon et al., 2003, 2008; Kurz et al., 2010, 2015; Wei et al., 2011a,b; Kembro et al., 2013; Glancy et al., 2015). As a consequence of these findings, the current paradigm, in which the individual mitochondrion is regarded as a separate and passive energy producer, may have to shift to a view of mitochondria as active and interconnected participants in a network regulating the whole cell energy metabolism *vis-à-vis* fluctuations due to combinations of amplitude modularity (AM) and frequency modularity (FM) modes. Along this line, evidence is accumulating suggesting that the FM mode of ROS regulation is uniquely suitable to regulate signals under physiological conditions (Gong et al., 2014, 2015; Ying et al., 2016), whereas under extreme conditions or strong stress, the FM mode activity becomes uncontrolled and significantly augments global ROS production (Zorov et al., 2000; Aon et al., 2003; Wang et al., 2008; Ying et al., 2016).

### Macroscopic UPE Fluctuations

The new paradigm may also have consequences for the emission of ultra-weak photon emission of living tissues. The dynamic 10–15 s superoxide flashes demonstrated by mitochondrial-targeted superoxide-specific fluorescent markers have raised the suggestion that these flashes should not only result in similar ROS signals but also in similar bursts of spontaneous ultra-weak photon emission. However, at microscopic scale such flash of UPE from single mitochondrion is far too weak to be detected, but based on the observed synchronization of superoxide flashes in some tissues, the amplitude of spontaneous UPE from synchronized ROS, may be detectable by “digitization” of spontaneous photon production, at least if synchronization extends over a macroscopic spatial area. The latter may be derived from UPE studies of human skin because histological studies have shown that its epidermal cells are organized in a thin layer parallel to the skin surface nourished by fluid, which penetrates the intercellular spaces from capillaries in the underlying connective tissue; the organization is comparable to a multi-layer of cultured cells upon a collagen matrix. Skin allows non-invasive measurement by placing a photomultiplier perpendicular upon the skin surface. Using sensitive technology including strict measurement protocols, a search for macroscopic fluctuations in UPE from skin resembling the 10–15 s during mitochondrial flashes may be demonstrated.

A few research teams constructed facilities for non-invasive multi-site UPE recording from human skin using a sensitive photomultiplier tube that could be manipulated over a human body in order to search for skin locations with high emission resulting in a maximal signal/noise ratio (Cohen and Popp, 1997; Van Wijk et al., 2014). Next to technological issues, a careful protocol for handling human participants prior to measurement was stepwise constructed, resulting in reliable measurements. Studies included a variety of body locations. The most extensive, topographical variation study consisted of 29 body sites (Van Wijk and Van Wijk, 2005). UPE from the hands and head were commonly higher than emissions from other body locations. Right-left symmetry of UPE was common, but dorsal-ventral symmetry could not always be observed. Furthermore, data suggested a common human anatomical UPE pattern. The existence of such pattern was first confirmed with a highly sensitive charge-coupled device (CCD) imaging system (Kobayashi, 2003) followed by a systematic quantification by the photomultiplier system (Van Wijk et al., 2006). Data also confirmed that the maximal signal/noise ratio was found for the dorsal and palm sides of left and right hand. Additional spectral decomposition studies of the emission of hands showed a major spontaneous emission at 470–570 nm, suggesting UPE was ROS related (Van Wijk and Van Wijk, 2005; Tsuchida et al., 2019). The conclusion from the macroscopic UPE studies – technological and methodological – was that emission of hand dorsal and palms are most likely for analysis of UPE signals for fluctuations on 10–15 s time scales (Van Wijk and Van Wijk, 2006).

The concept that photons may be part of an integrated mitochondrial organization of biochemical reactions with “frequency-modulatory (FM) modes” and “amplitude-modulatory (AM) modes”, and additionally, the measured quantity of photon counts is a rare event in short time of binning, has put emphasis on the Poisson distribution of photon count (Kobayashi et al., 1998; Kobayashi and Inaba, 2000; Van Wijk et al., 2010; Bajpai et al., 2013). Poisson distribution is characterized by only one parameter, mean, because variance is equal to mean. Fano (1947) pointed out that the ratio of variance to mean is an indicator of the deviation of observed values from a Poisson distribution. This ratio is called Fano factor. If the ratio is greater than one, it is called super-Poissonian and if the ratio is less than one, it is called sub-Poissonian. Super-Poisson distribution of photon counts implies bunching of emitted photons, indicating clustering of excitation processes. Similarly, the opposite phenomenon of anti-bunching of photons indicated by a sub-Poisson distribution of photon counts may be initiated under specific conditions of excitation feedback. It has been utilized for characterizing spontaneous photon emission of bacteria (Kobayashi et al., 1998) and *Dictyostelium* (Kobayashi and Inaba, 2000). Studies have taken careful corrections for background noise of the photomultiplier (Poplová et al., 2017).

To utilize Fano factor analysis of the human skin, UPE signal for extraction of information about photon clustering within time periods of 10–15 s means Fano factor estimations within different measuring time windows of sufficiently long recordings of sequenced events. It also requires checking that an upward or downward trend in the signal is absent plus that the information is in the sequence (Van Wijk et al., 2005). Using these precautions, Fano factor dependence on window time (*T*), from a minimum duration of 50 ms up to a maximum of 15 s, the Fano factor time curve were estimated for UPE signals measured at four hands locations of 50 male subjects (Van Wijk et al., 2010; Bajpai et al., 2013). The analysis demonstrated that the Fano factor of true signal increased when the window size rises above 6 s; Fano factor values of 15 s, window size for individual hand locations were spread over a large region. The data also indicated that the *F*(*T*) shape is essentially a combination of a small sub-population of individuals with predominantly sub-Poissonian and a larger sub-population with predominantly super-Poissonian photo count distribution. The physiologic significance of such signals was estimated by calculating any relationship between hand locations. The distribution of the values of the Fano factor in the signals from left and right hand is nearly the same. The correlation coefficient between the Fano factor data of left‐ and right-hand showed that they were significantly correlated. The Fano factor of the palm and dorsal locations was also correlated.

To conclude section Enhanced Integration: Mitochondrial Dynamics (2000–2020), it can be stated that we have reached the dome in our light house metaphor (Figure 1) with its major characteristic: dynamic signaling. This dynamics includes the most recent technological advances: (1) the introduction of confocal scanning and deconvolution microscopy for the visualization of the fusion and fission dynamics of the mitochondrial network; (2) the introduction of mitochondrial function sensitive fluorescent substances for measuring spatial and temporal superoxide flash properties; and (3) the introduction of technology and methodology for non-invasive recording of UPE dynamics of high-emitting topographical regions of human skin, allowing analysis of photon count distributions. It is also evident that some suggestions linking different dynamic aspects of mitochondria need verification: (1) relation between structural dynamics and functionality of mitochondria (at microscopic scale) and (2) relation between synchronized (bunching of) photons emitted from a large spatial skin area and the ROS events and microscopic scale. For both, there is no absolute evidence. This needs additional experimental studies. This perspective follows in the next, final section.

## Perspective

Like mitochondria, research itself shows the process of fission and fusion. Fission in research development commonly parallels increased specialization, and fusion is the integration of separate fields to increase the intrinsic value. Our review has focused on the fascinating integration of three independent piles of knowledge during the last six decades within the field of mitochondrial research. It combined the mitochondria as ATP producers (power houses), ROS producers (causing damage), and as sources of UPE. More than any previous review, it has emphasized the possible role of UPE in increasing our understanding of mitochondrial biology. This has been reflected in the metaphor of the lighthouse (Figure 1), and in its present state as the dynamically pulsing dome. This consists of the most important advances resulting from the discovery of mitochondrial flash activity: the control of intracellular metabolism by typical AM-FM modes at the individual mitochondrion level and the spontaneous UPE fluctuations resulting from synchronized AM-FM modes of ROS in dynamic mitochondrial energetics dynamics at macro-scale, and at organ (skin) level.

The perspective of this integration process is the application of non-invasive and minimally invasive technologies to measure mitochondrial (dys)function (Zhao et al., 2016; Sun et al., 2017). However, it is evident that many questions need to be answered. Mitochondria are not all equal but are specialized across organs in their functions ensuring that they are not only functionally matched to the demand of the organ, but also to the body as a whole. Furthermore, mitochondria may exhibit qualitative physiological differences in some disease states. With more studies on mitochondrial flash activity consisting of the design of new generations of indicators to dissect the biology of the mitochondrial flash activity and the development of advanced technology for recording non-invasive ultra-weak photon emission and signal pattern recognition as an *in vivo* biomarker for mitochondrial function, new features and regulatory mechanisms are likely to be discovered, which may advance diagnostics with respect to diseases and aging. Progress in mechanistic understanding not only may yield new diagnostic opportunities but may also enable the testing of health effects of targeted therapeutic and preventative strategies: physical activity, diet, and other lifestyle measures. Together, these future lines of research will provide vital contributions for moving the integration of ROS, UPE, and stress forward beyond 2020 and expand the relevance of mitochondria in medicine.

## Author Contributions

RW and EW conceived the manuscript. RW, EW, JP, MY, YY, and JH participated in writing the manuscript. All authors contributed to the article and approved the submitted version.

### Conflict of Interest

The authors declare that the research was conducted in the absence of any commercial or financial relationships that could be construed as a potential conflict of interest.
